# Clinical and radiographical analysis of percutaneous kyphoplasty with multi-point cement anchoring technique for preventing bone cement displacement in Kümmell’s disease of stage I and II

**DOI:** 10.3389/fendo.2025.1538337

**Published:** 2025-06-11

**Authors:** Heng Wu, Xiao Dai, Hao Liu, Xiang Yang, Shenao Liu, Shuang Xu, Hao Chi, Yuquan Chen, Song Wang

**Affiliations:** ^1^ Department of Orthopedics, The Affiliated Hospital of Southwest Medical University, Luzhou, China; ^2^ Clinical Medical College, Southwest Medical University, Luzhou, China; ^3^ School of Public Health and Preventive Medicine, Faculty of Medicine, Nursing & Health Sciences, Monash University, Melbourne, Australia

**Keywords:** osteoporosis, Kümmell’s disease, percutaneous kyphoplasty, multi-point cement anchoring technique, bone cement displacement

## Abstract

**Background:**

The present study introduced a novel technique called percutaneous kyphoplasty with multi-point cement anchoring technique (A-PKP) to prevent bone cement displacement in patients with stage I and II Kümmell’s disease (KD).

**Methods:**

A total of 82 patients with stage I and II KD were treated with PKP in our hospital from April 2020 to October 2022. The patients were divided into two groups: A-PKP group (N=39) where the Kirschner needle was used for the multi-point cement anchoring technique, and conventional transverse process-pedicle percutaneous kyphoplasty group (T-PKP group; N=43) where the Kirschner needle was not used. The operation time, volume of cement, VAS score, ODI score, cement distribution pattern and score, bone cement leakage, adjacent vertebra fracture, and bone cement displacement were compared between the two groups. A logistic regression model was used to evaluate the association between outcome variables and adjacent vertebral fractures, as well as to identify potential protective and risk factors following kyphoplasty.

**Results:**

All patients in both groups were operated successfully, with no serious complications reported. Compared with T-PKP patients, A-PKP patients had longer operation time (39.7 ± 4.86 min vs. 34.5 ± 3.18 min, P < 0.05), greater volume of cement (5.1 ± 0.41 ml vs. 4.3 ± 0.27 ml, P < 0.05), greater improvement in Visual Analog Scale (2.0 ± 0.48, 1.92 ± 0.72 vs. 3.0 ± 0.10, 3.1 ± 0.62, P < 0.05) and Oswestry Disability Index scores (17.9 ± 2.38, 14.8 ± 2.02 vs. 20.2 ± 3.31, 17.2 ± 2.55, P < 0.05) during follow-ups, more spongy cement configuration with higher distribution scores (10.0 ± 1.17 vs. 7.74 ± 1.08, P < 0.05), lower incidence of bone cement leakage (20.5% vs. 27.9%, P > 0.05), and lower rate of adjacent vertebra fractures (5.1% vs. 18.6%, P < 0.05) and bone cement displacement (2.5% vs. 20.9%, P < 0.05). The logistic regression results reveal that bone cement distribution score (OR= 0.355, 95% CI 0.171–0.734, P=0.005) acts as protective factor of adjacent vertebral fractures following kyphoplasty.

**Conclusion:**

The A-PKP technique appears to be a safer and more effective alternative for patients with stage I and II KD. It effectively alleviates pain, enhances cement diffusion, and minimizes the risk of bone cement displacement compared with the T-PKP.

## 1 Introduction

Kümmell’s disease (KD) is a condition characterized by delayed posttraumatic vertebral collapse with vertebral osteonecrosis, which occurs in approximately one-third of patients with osteoporotic vertebral compression fractures (OVCF) (1, 2). First described by Dr. Hermann Kümmell in 1893, KD has become increasingly prevalent as the population is aging (3). KD typically presents as progressive, painful kyphosis that develops after an asymptomatic phase lasting weeks to months following minor spinal trauma. In severe cases, patients may develop paraparesis, or partial (2–4). The intervertebral vacuum cleft (IVC), along with marginal sclerosis, is a hallmark radiological feature of KD and indicates vertebral ischemic necrosis and the formation of a dynamic unstable pseudarthrosis (5, 6). Hasegawa et al. (7) demonstrated that the IVC corresponds to a pseudarthrosis, where the fracture site is surrounded by thick fibrocartilaginous tissue, which is indicative of unhealed bone and may obstruct penetration of bone cement.

Conservative treatments for KD, such as bed rest, nonsteroidal anti-inflammatory drugs, and back bracing, are generally ineffective because they do not address the underlying bone necrosis or halt the progression of kyphosis (8, 9). For stage I and II KD patients, percutaneous kyphoplasty (PKP) is considered a minimally invasive surgery that can alleviate pain by restoring vertebral height, stabilizing the spine, and correcting kyphosis (10, 11). However, traditional PKP, such as transverse process-pedicle approach PKP (T-PKP), encounters challenges because of the presence of dense fibrocartilaginous tissue surrounding the IVC (12). This issue impedes the appropriate penetration of the infused bone cement, typically polymethyl-methacrylate (PMMA), into the trabecular bone (7). This results in inadequate cement interdigitation, leading to a blocky cement pattern, which can increase the risk of postoperative cement displacement and potentially result in surgical failure surrounding trabecular bone, leading to the formation of a blocky cement pattern (5, 13, 14). In response to these limitations, Qin et al. (15) introduced a unilateral anchoring technique during percutaneous vertebroplasty (PVP) for treating neurologically intact KD patients and yielded satisfactory results by improving cement anchoring and stability. However, this unilateral PVP anchoring technique did not adequately disrupt the fibrocartilaginous membrane surrounding the IVC, resulting in suboptimal cement distribution and insufficient anchoring points.

In this study, we employed simple curved-tipped Kirschner needles to perforate and create multiple puncture sites in dense fibrocartilaginous membrane before cement injection and termed this technique as PKP with multi-point cement anchoring (A-PKP). We hypothesized that A-PKP would provide superior cement distribution and reduce cement displacement compared to T-PKP.

## 2 Materials and methods

### 2.1 Patients

This single-center retrospective study was approved by the Ethics Committee of the Affiliated Hospital of Southwest Medical University (KY2023328). All methods and procedures adhered strictly to relevant guidelines and regulations, with informed consent being obtained from all participants. In total, 82 patients diagnosed with stage I and II KD were treated at hospital and followed up between April 2020 and October 2022. These patients, diagnosed with stage I and II KD based on clinical and radiographical presentations, were neurologically intact with compressed vertebrae showing low signal on T1-weighted MRI, high linear signal with surrounding low intensity on T2-weighted and STIR sequences MRI, along with IVC and an intact posterior vertebral wall. A power analysis was performed prior to the study using G*Power, with an effect size of 0.8 (large effect size), a significance level of 0.05, and a desired power of 0.80. The required sample size was estimated to be 52 participants (26 per group). Based on the surgical approach determined by the treating surgeon, guided by clinical judgment and individual patient factors, patients were divided into two groups: A-PKP group (N=39) where the Kirschner needle was used for the multi-point cement anchoring technique, T-PKP group (N=43) where the Kirschner needle was not used. The postoperative management was identical in both two groups.

### 2.2 Selection criteria

Inclusion criteria: The study included patients who (1) had back pain following minor trauma and were diagnosed with stage I and II KD (11); (2) had a bone mineral density (BMD) T-score of < −2.5; (3) were confirmed to have single-segment KD with an intact posterior vertebral wall; (4) exhibited consistency between the “responsible” vertebra identified in physical and imaging examinations; and (5) received unilateral A-PKP or T-PKP and were followed up for at least 12 months.

Exclusion criteria: patients with (1) nerve injury symptoms or posterior wall defects (stage III KD); (2) primary or metastatic spinal tumors, spinal tuberculosis, and spinal infections; (3) severe vertebral body compression, which rendered puncture impossible; (4) patients with local or systemic infections that affected the operation; and/or (5) coagulation dysfunction and other conditions that made surgery intolerable were excluded from the study.

### 2.3 Surgical procedures

#### 2.3.1 A-PKP

**Figure 1:**
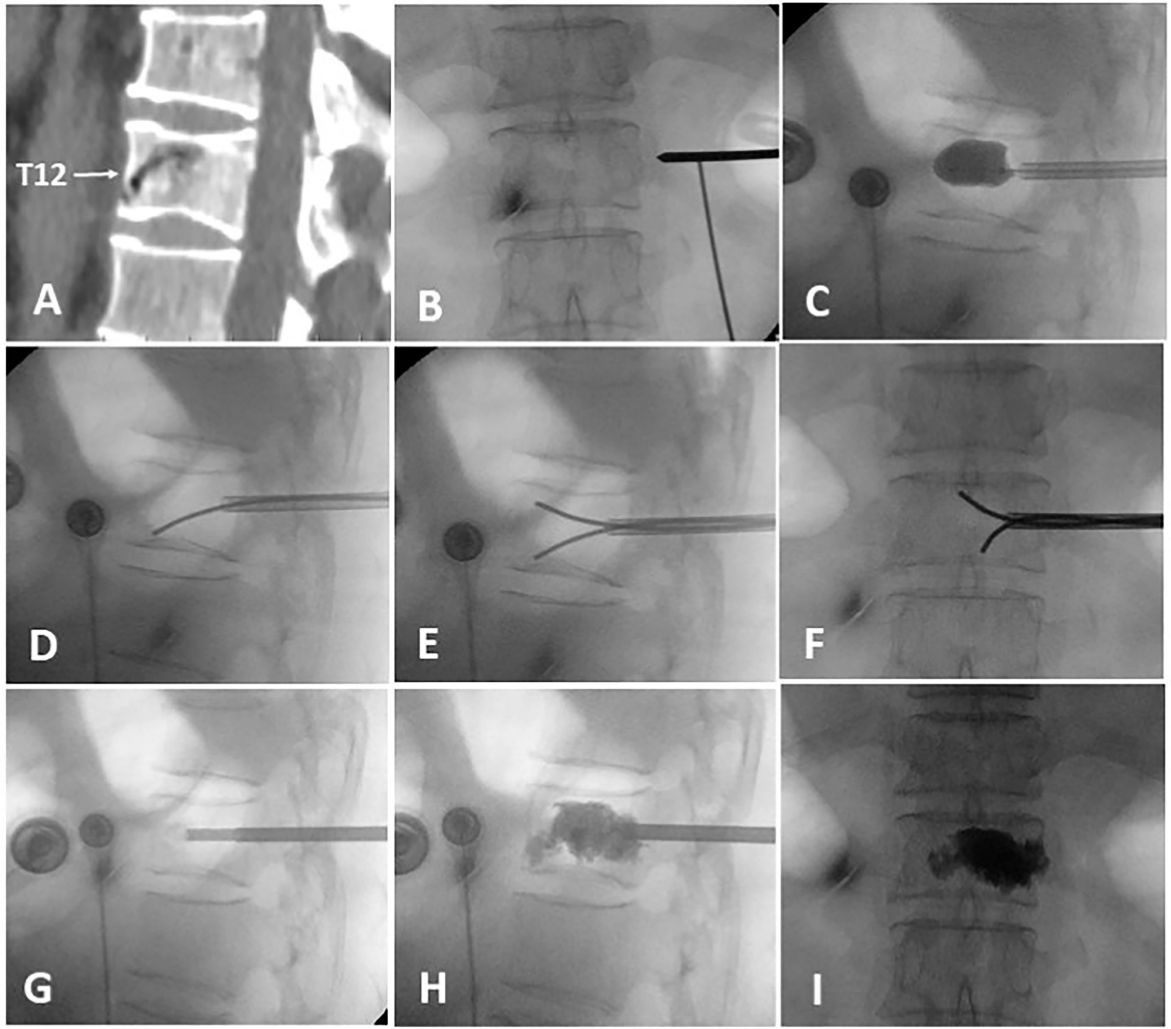
The intraoperative process of A-PKP. **(A)** Preoperative sagittal CT showing IVC at T12 with cortex defect. **(B)** TPA puncture approach. **(C)** Balloon used for fracture reduction. **(D-F)** Curved-tipped Kirschner needles puncturing the fibrocartilaginous membrane towards the upper and lower endplates. **(G)** 1ml of late-phase wire-drawing cement blocking the cortical defect. **(H, I)** Multi-point anchoring pattern created by interlocking cement with the cancellous bone through the punctured fibrocartilaginous membrane.

The surgery was performed under local infiltration anesthesia, with continuous monitoring of vital signs throughout the procedure. Real-time observation of the patient’s lower limb sensation and movement was conducted to ensure no spinal cord compression. The patient was placed in a prone, hyperextended position, with the abdomen suspended in the air. The puncture point of the transverse process-pedicle approach (TPA) was located at the midline of the transverse process, 3-5mm to the unilateral pedicle margin surface projection (12). The operation area was routinely disinfected and covered with a sterile towel. Subsequently, a puncture needle was advanced into the vertebra under fluoroscopic guidance, and positioned and inserted within the IVC, aiming for two-thirds of the anterior region. After reaching the target area, a guide needle, dilation cannula, and working cannula were sequentially introduced under fluoroscopic guidance. A balloon was implanted to restore vertebral body height and withdrawn upon contact with upper or lower endplate. A curved-tipped Kirschner needle was inserted through the working cannula into the IVC under lateral C-arm fluoroscopic guidance. The direction of the needle was controlled to puncture toward the lower endplate. Simultaneously, another curved-tipped Kirschner needle was inserted and directed toward the upper endplate. Subsequently, these needles were used to puncture and disrupt the thick fibrocartilaginous membrane surrounding the IVC by repeatedly moving the needles in and out, creating multiple puncture points. To block any defect in the anterior cortex, 1ml of late-phase wire-drawing cement was first injected. Around 3-4ml of early-phase wire-drawing cement was gradually injected into the IVC. Through puncture points made in the fibrocartilaginous membrane, bone cement was diffused into the surrounding cancellous bone until it contacted endplate. Once contact with the endplate was confirmed, the work cannula was retracted by 0.5 cm, and an additional 1 ml of late-phase wire-drawing cement was injected for anchoring. The injection created a multi-point anchoring pattern by interlocking the cement with the cancellous bone structure through the punctured fibrocartilaginous membrane (**Figure 1**).

#### 2.3.2 T-PKP

The puncture and cement injection methods used in the T-PKP group were similar to those in the A-PKP group. However, the T-PKP group did not employ curved-tipped Kirschner needles to disrupt the fibrocartilaginous membrane.

### 2.4 Postoperative management and follow-up

After surgery, the patients were instructed to rest in bed for 1 day followed by a treatment regimen to strengthen bones, including calcium carbonate, vitamin D3 tablets, and zoledronic acid injection (16, 17). Subsequently, the patients were encouraged to begin daily activities and functional exercises under supervision, while wearing a back brace, before discharge. Follow-up assessments were conducted at 3 days postoperatively and the final follow-up (≥12 months).

### 2.5 Clinical and radiological evaluation

The operation time, volume of bone cement injected, cement distribution, and incidence of cement leakage were recorded. During the procedure, C-arm fluoroscopy was used to monitor the cement injection process. The injection was stopped when cement symmetrically occupied the anterior three-quarters of the vertebral body, reached the posterior one-quarter, made contact with the superior and inferior endplates, or when leakage occurred (18, 19). Clinical and radiological assessments were performed before the surgery, 3 days after surgery, and at the final follow-up. Pain relief and function recovery were assessed using the VAS and ODI scores. All VAS and ODI scores were assessed by two experienced spine surgeons, with the final result being the average value, to ensure the objectivity and accuracy of the scoring process.

**Figure 2:**
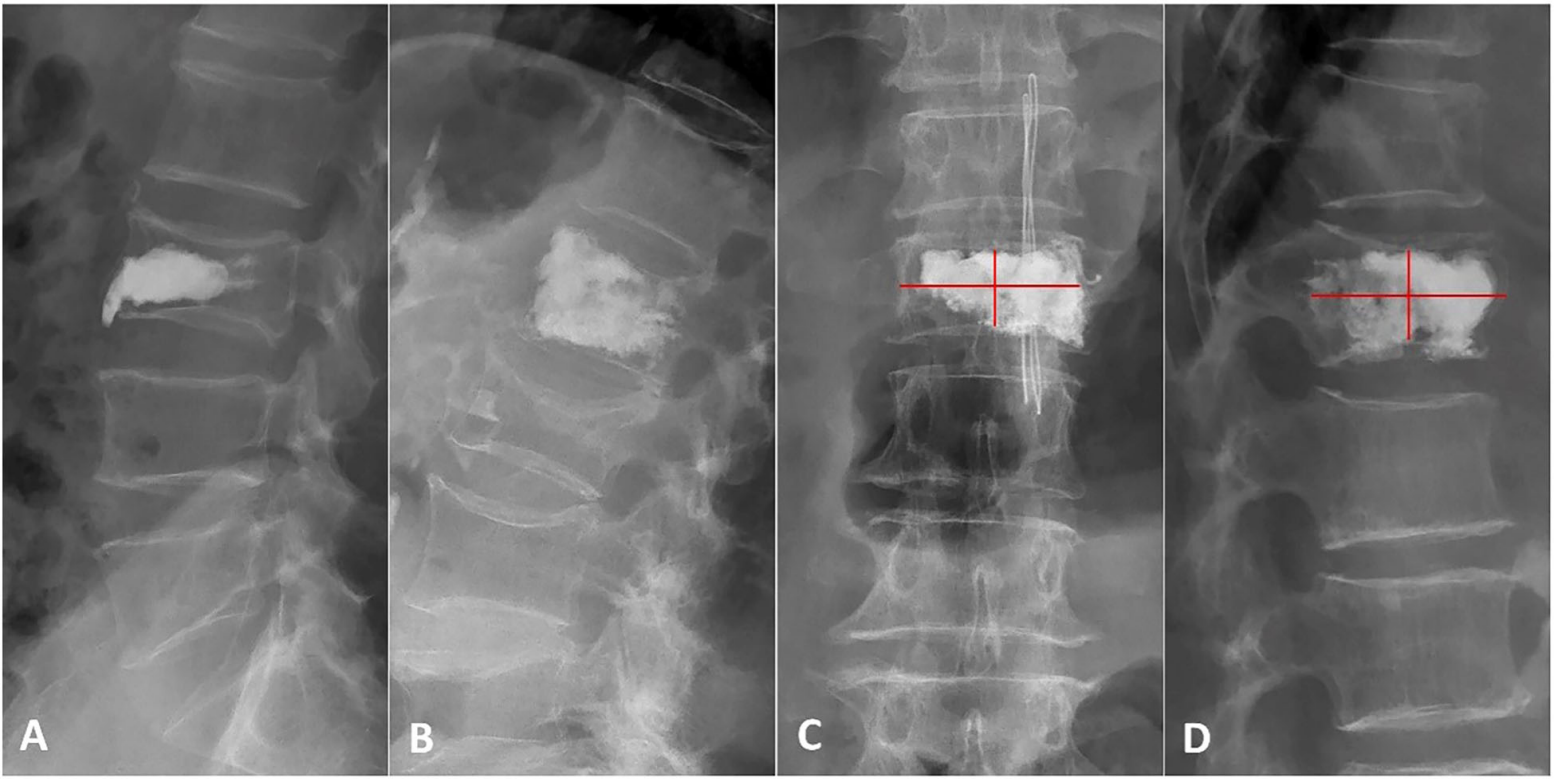
Patterns and scores of bone cement distribution. **(A)** Lateral X-ray showing a blocky cement pattern. **(B)** Lateral X-ray showing a spongy pattern. **(C, D)** Anteroposterior and lateral X-ray using the 12-score method: the vertebra is divided into four quadrants. 2 scores are counted if the bone cement filling exceeds one-third of each quadrant, bone cement contacted the upper or lower endplate or bone cement crossed the middle line.

The qualitative and quantitative aspects of cement distribution were documented. Qualitatively, bone cement distribution was classified into two patterns based on X-ray: spongy (**Figure 2A**) or blocky (**Figure 2B**) (13). A 12-point scoring method was used to assess cement distribution quantitatively (20). The vertebra was divided into four quadrants on both anteroposterior and lateral X-rays. A quadrant was considered effective if the cement filling exceeded one-third of its area, with each effective quadrant receiving 2 points. Additionally, if the cement contacted the upper or lower endplate on the lateral X-ray, or if the cement crossed the midline on either the anteroposterior or lateral X-rays, each of these signs was also considered an effective quadrant, earning 2 points (**Figures 2C, D**). The total score ranged from 0 to 12 points, with higher scores reflecting better cement distribution through the vertebra.

**Figure 3:**
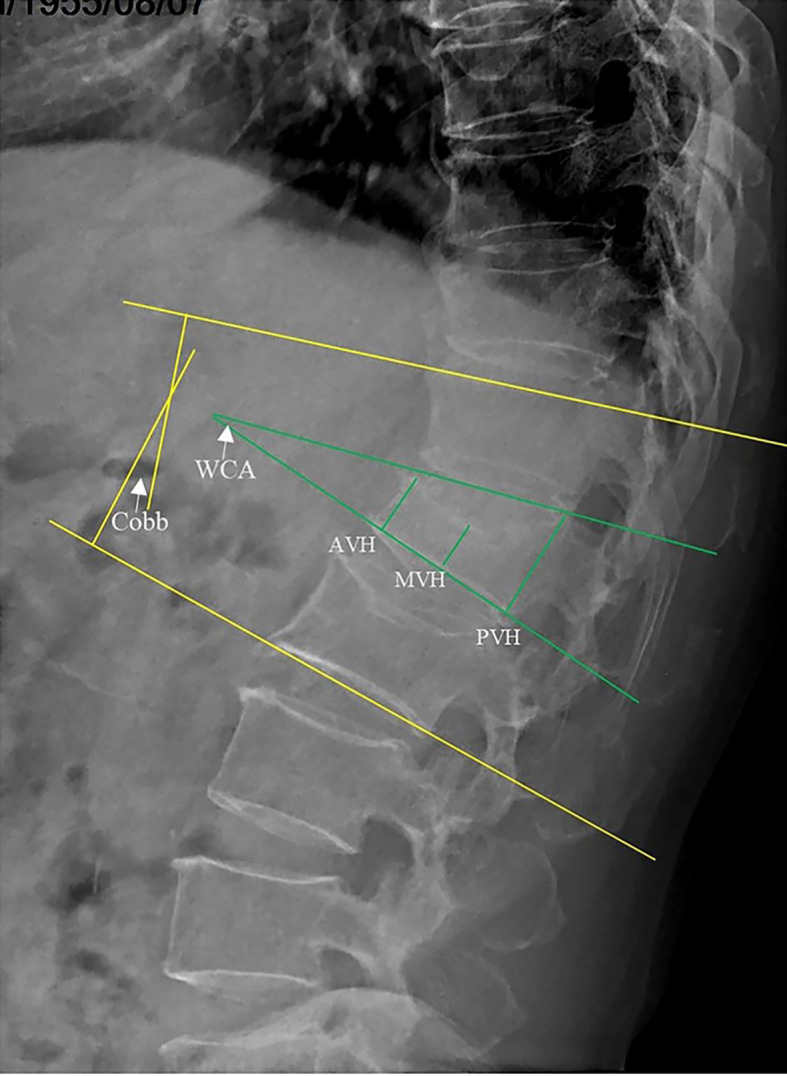
Radiographic measurements on lateral X-ray: Anterior vertebral height (AVH), middle vertebral height (MVH), posterior vertebral height (PVH), Cobb’s angle (Cobb), and wedge-shaped Cobb’s angle (WCA).

Anterior vertebral height (AVH), middle vertebral height (MVH), posterior vertebral height (PVH), Cobb angle, and wedge-shape Cobb angle (WCA) were measured to assess the improvement in vertebral height and kyphosis before the operation, 3 days after the operation, and at final follow-up (**Figure 3**). The AVH, MVH and PVH were defined as the perpendicular distances from the vertebral body’s anterior wall, the midpoint of the vertebral body, and the posterior wall to the horizontal lines drawn along the upper and lower endplates of the vertebrae on lateral X-ray. The Cobb angle was defined as the angle between the parallel lines drawn along the upper endplate of the vertebra above the fractured vertebra and the lower endplate of the vertebra below the fractured vertebra, as measured on lateral X-ray. All measurements were conducted by assessors aware of the treatment groups. To minimize bias and error, multiple independent measurements were taken, averaged, and evaluated using standardized criteria. Assessors were trained, and inter-rater reliability was ensured through consensus in case of disagreement. Complications such as bone cement displacement and adjacent vertebra fracture were analyzed during follow-ups. Bone cement displacement was identified by anterior cortical rupture and forward migration of the cement on lateral X-ray (14). Adjacent vertebra fracture was suspected based on vertebral height collapse observed on X-rays and confirmed by bone marrow edema with high signal intensity on STIR MRI sequences. Additionally, bone cement leakage was initially suspected based on the observed presence of extravertebral material during the procedure and was subsequently confirmed through postoperative three-dimensional CT imaging.

### 2.6 Statistical analysis

Data were analyzed using SPSS software version 29.0. Normally distributed continuous variables were presented as mean ± standard deviation, while nominal variables were reported as frequencies or proportions. Intragroup comparisons of continuous variables before and after the operation were conducted using paired-sample t-tests. The independent-sample t-test and Mann–Whitney U test were performed to compare continuous parameters between the A-PKP and T-PKP groups. Proportional differences were examined using the Chi-square test. A logistic regression model was used to evaluate the association between outcome variables and vertebral fractures, as well as to identify potential protective and risk factors following kyphoplasty. Statistical significance was defined as P < 0.05.

## 3 Results

### 3.1 General characteristics of the patients

**Figure 4:**
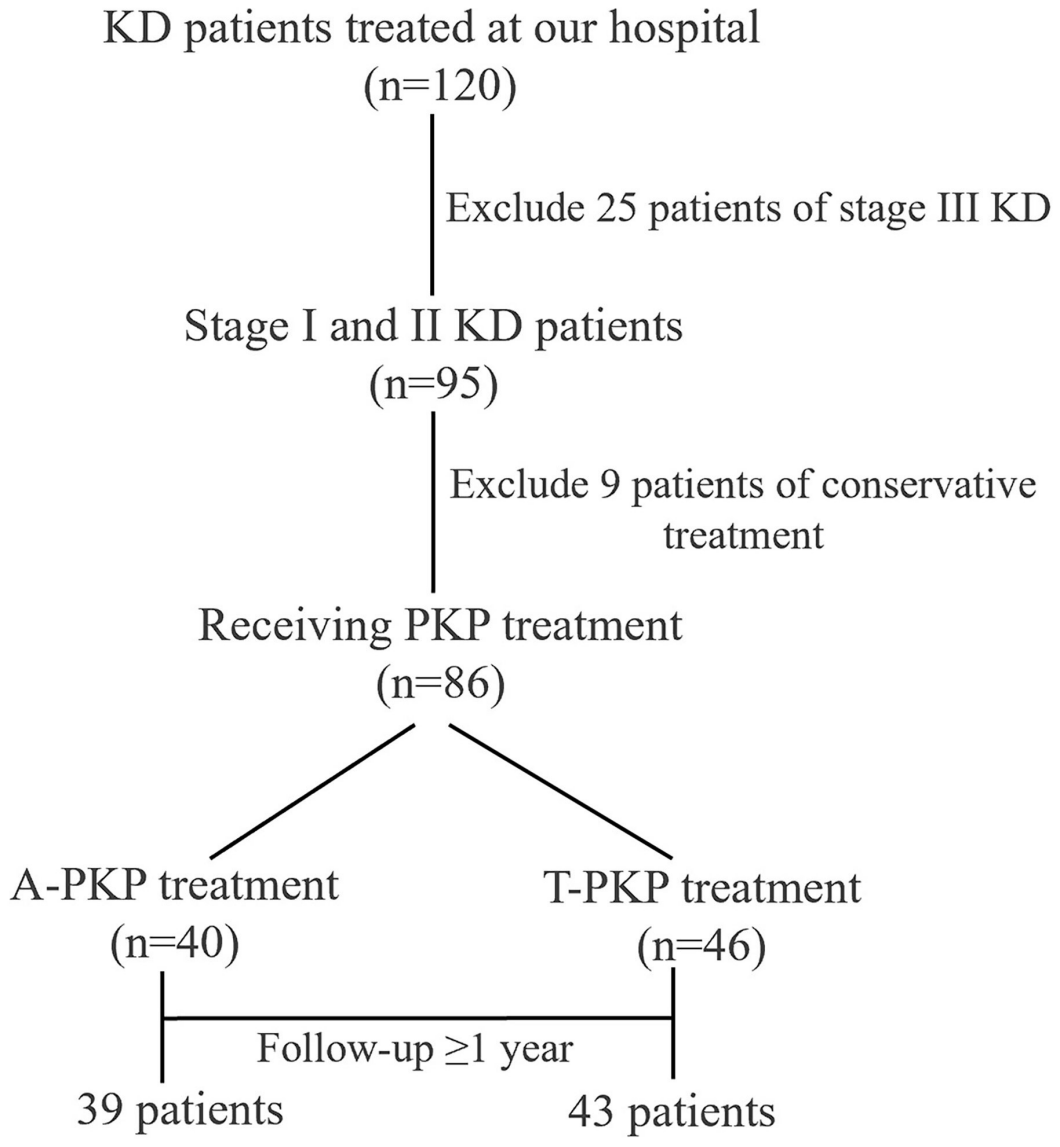
Flow diagram of patient selection process.

**Figure 5:**
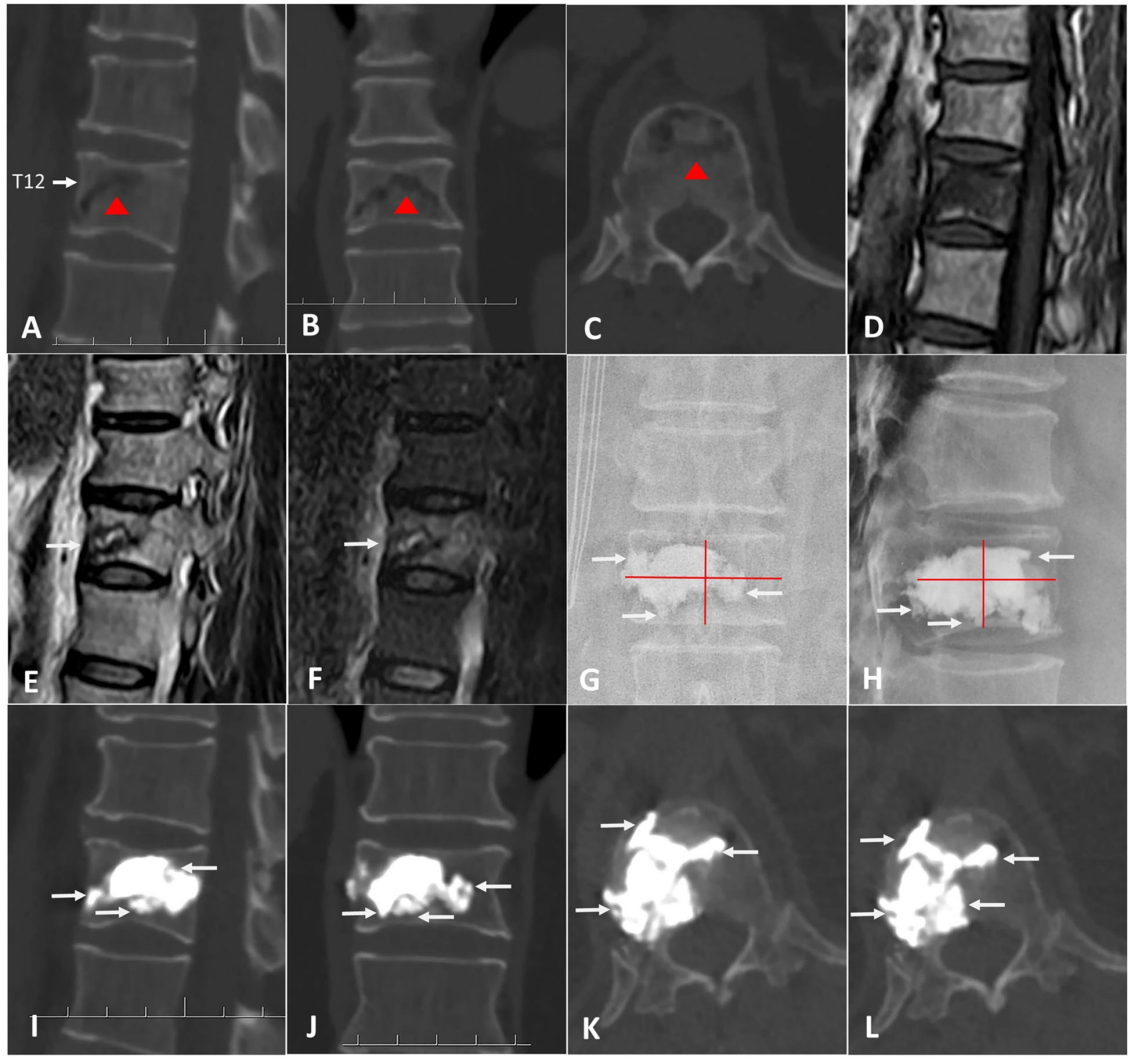
A 74-year-old man with KD at T12 was treated by A-PKP. **(A-C)** Preoperative CT showing IVC (red triangle). **(D)** the sagittal T1-weighted MRI showing low signal intensity in IVC. **(E, F)** the sagittal T2-weighted and STIR sequences MRI showing high signal with surrounding low intensity (Double-line sign; DLS). **(G, H)** postoperative X-ray exhibiting spongy cement pattern. **(I-L)** CT scans at final follow-up showing satisfactory cement anchorage (white arrows).

**Figure 6:**
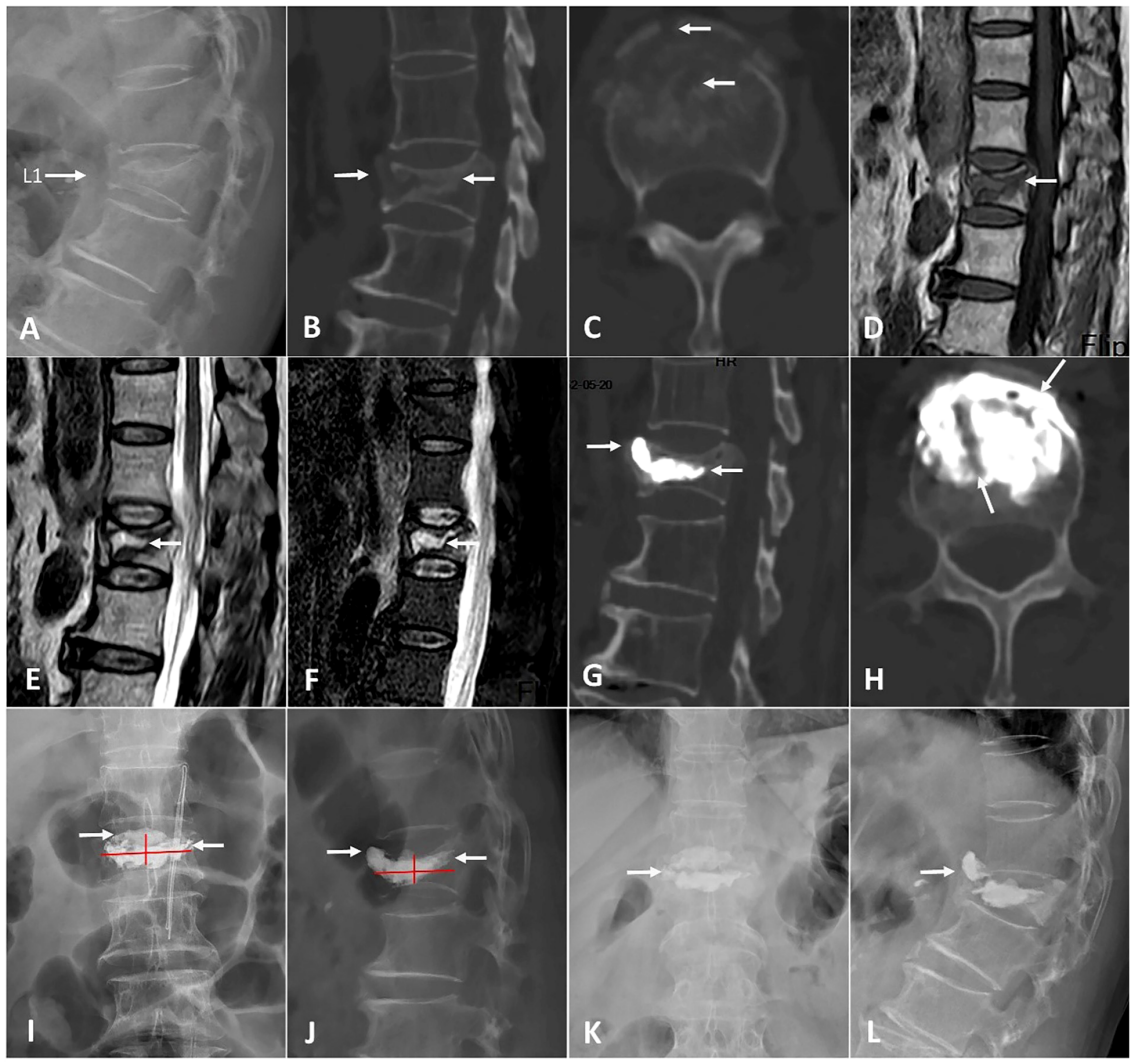
A 70-year-old woman with KD at L1 was operated by T-PKP. **(A-C)** Lateral X-ray and CT showing an anterior cortex defect and IVC (white arrows). **(D)** The sagittal T1-weighted MRI exhibiting a low signal intensity in IVC. **(E, F)** The sagittal T2-weighted and STIR sequences MRI exhibiting a high signal with a surrounding low intensity (double-line sign; DLS). **(G-J)** Postoperative CT and X-ray displaying a blocky cement pattern. Cements are separated by a fibrocartilaginous membrane in picture **(H)** (white arrows). **(K, L)** Anteroposterior and lateral X-ray at a final follow-up, showing vertebral collapse [white arrow in picture **(K)**] and bone cement displacement [white arrow in picture **(L)**].

A total of 82 patients were enrolled in this study: 39 patients in the A-PKP group and 43 patients in the T-PKP group (**Figure 4**). All patients underwent successful surgery and were followed up between April 2020 and October 2022. The A-PKP and T-PKP groups exhibited no significant differences in terms of baseline characteristics, including sex, age, disease duration, BMD, disease stage, AVH, Cobb angle, anterior cortex defect, and follow-up duration (**Table 1**; P > 0.05). These findings indicate that both groups were comparable in baseline characteristics. **Figures 5**, **6** present typical cases of both A-PKP and T-PKP techniques.

**Table 1:** Baseline date of two groups.

Parameters	A-PKP	T-PKP	t/χ2	p
Cases	39	43		
Male/Female	9/30	15/28	1.378	0.241
Age (years)	73.3 ± 6.55	74.0 ± 5.95	-0.446	0.657
Course of disease (months)	5.4 ± 2.00	5.7 ± 1.79	-0.730	0.468
BMD (T value)	-3.8 ± 0.74	-3.8 ± 0.63	0.343	0.732
Stage (I/II)	17/22	24/19	1.222	0.269
Injured segment				
T8 (cases)	0	1		
T9 (cases)	1	3		
T10 (cases)	3	3		
T11 (cases)	1	1		
T12 (cases)	13	14		
L1 (cases)	18	15		
L2 (cases)	2	5		
L3 (cases)	0	1		
L4 (cases)	1	0		
Anterior vertebral height (mm)	14.5 ± 5.23	13.9 ± 4.16	0.528	0.599
Cobb angle (°)	20.3 ± 7.54	20.2 ± 8.12	0.080	0.936
Anterior cortex defect (Yes/No)	19/20	14/29	2.221	0.136
Follow-up time (months)	18.4 ± 2.2	18.6 ± 2.7	-0.358	0.721

### 3.2 Intraoperative, clinical, and complication outcomes

The incidence of bone cement leakage was higher in the T-PKP group than in the A-PKP group (20.5% vs.27.8%), but this difference was not statistically significant (P > 0.05). The operation time was longer in the A-PKP group than in the T-PKP group (39.7 ± 4.86 min vs. 34.5 ± 3.18 min, P < 0.05). Additionally, the A-PKP group used more bone cement than the T-PKP group (5.1 ± 0.41 ml vs. 4.3 ± 0.27 ml, P < 0.05). The spongy distribution pattern was higher in the A-PKP group (35 cases, 89.7% vs. 12 cases, 27.9%, P < 0.05). Furthermore, the A-PKP group had a higher bone cement distribution score than the T-PKP group (10.0 ± 1.17 vs. 7.74 ± 1.08, P < 0.05) (**Table 2**).

**Table 2:** Comparison clinical outcomes of two groups.

Parameters	A-PKP	T-PKP	t/χ2	p
VAS (scores)				
Preoperative	8.0 ± 0.49	8.0 ± 0.59	0.214	0.831
Postoperative 3 days	2.0 ± 0.48^*^	3.0 ± 0.10^*^	-7.650	<0.001
Final follow-up	1.92 ± 0.72^*#^	3.1 ± 0.62^*^	-8.136	<0.001
ODI (%)				
Preoperative	77.5 ± 3.39	76.2 ± 3.22	1.728	0.088
Postoperative 3 days	17.9 ± 2.38^*^	20.2 ± 3.31^*^	-3.552	0.001
Final follow-up	14.8 ± 2.02^*#^	17.2 ± 2.55^*#^	-4.670	<0.001
Operation time (min)	39.7 ± 4.86	34.5 ± 3.18	5.648	<0.001
Bone cement volume (ml)	5.1 ± 0.42	4.3 ± 0.34	9.908	<0.001
Spongy/Blocky cement pattern (case)	35/4	12/31		
Rate of Spongy pattern (%)	89.7	27.9	31.965	<0.001
Bone cement distribution (score)	10.0 ± 1.17	7.74 ± 1.08	10.016	<0.001
Bone cement leakage (%)	20.5	27.9	0.606	0.436
Yes (cases)	8	12		
No (cases)	31	31		
Adjacent vertebra fractures (%)	5.1	18.6	4.972	0.026
Yes (cases)	2	11		
No (cases)	37	32		
Bone cement displacement (%)	2.5	20.9	4.841	0.028
Yes (cases)	1	9		
No (cases)	38	34		

^*^Compared to pre-operative, p<0.05.

^#^Compared to postoperative 3 days, p<0.05.

**Figure 7:**
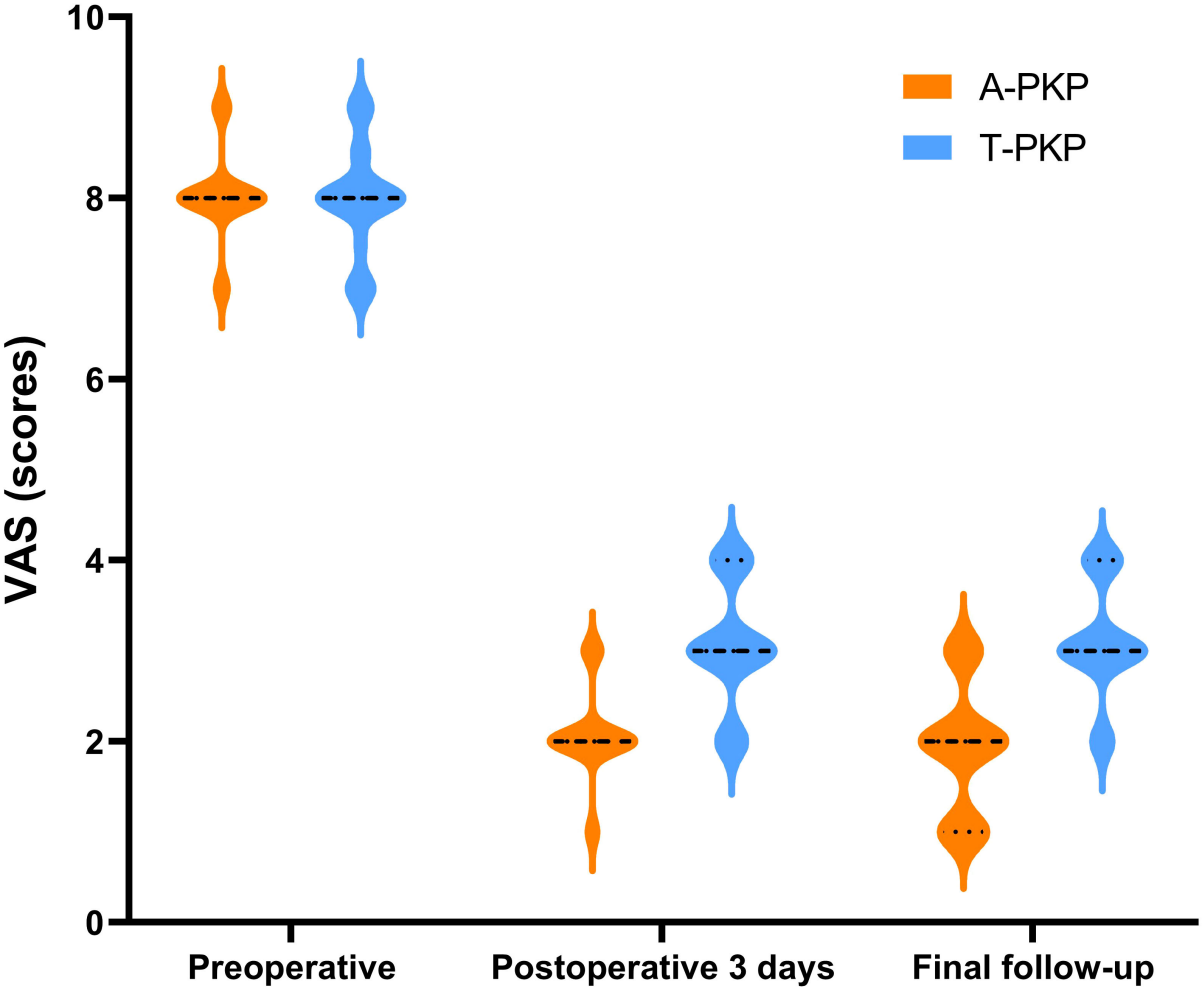
Histograms for VAS between the two groups.

**Figure 8:**
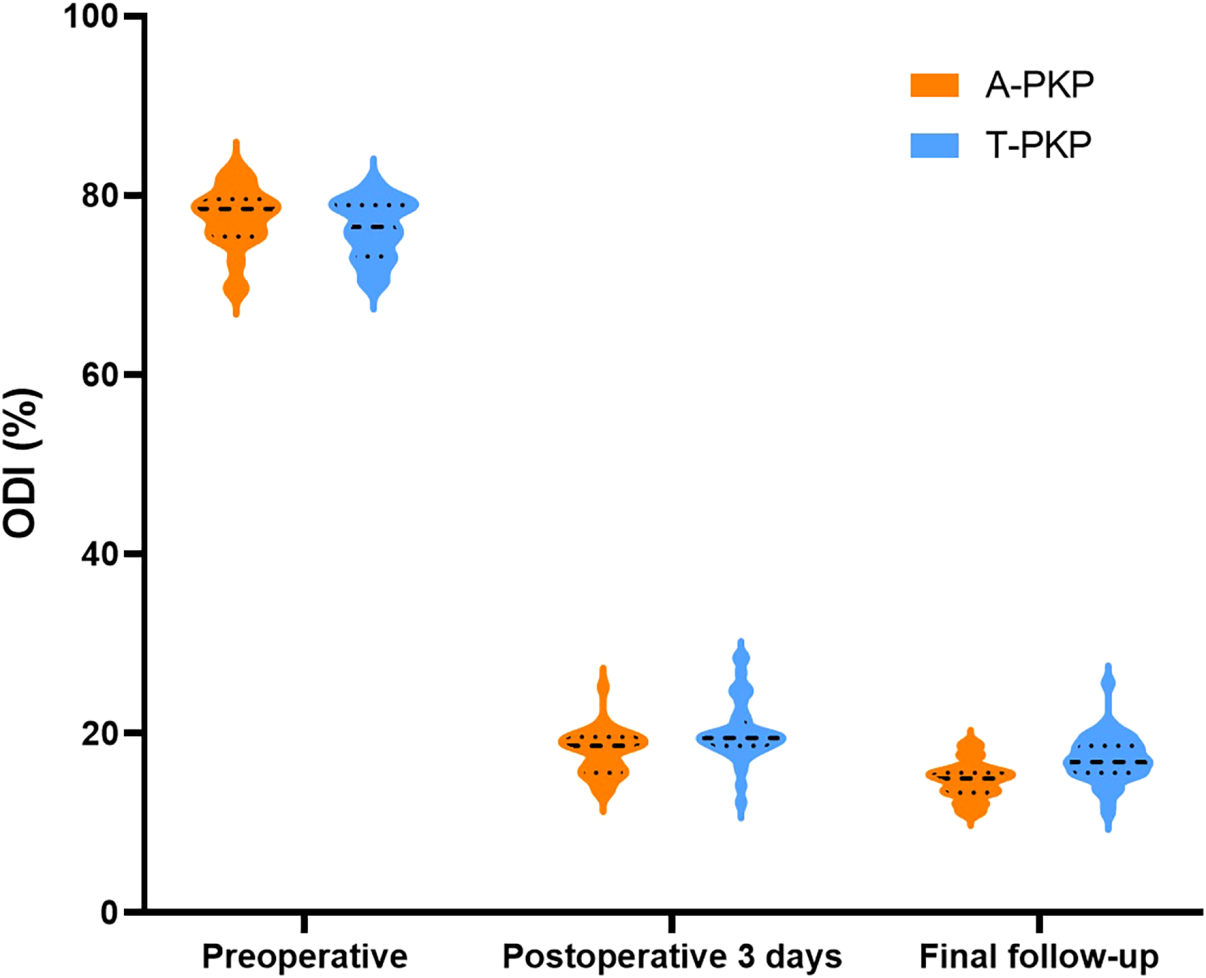
Histograms for ODI between the two groups.

Regarding clinical outcomes, both VAS and ODI scores exhibited no significant differences between the two groups preoperatively (P > 0.05). The difference score (Δ) was calculated by subtracting the preoperative score from the score at the final follow-up. Minimal clinically important differences (MCID) were defined based on previous studies: ΔODI of -9.5 and ΔVAS of -2.2 for pain (21). At subsequent follow-ups, both VAS and ODI scores reduced significantly in the A-PKP group (P< 0.05). In particular, the A-PKP group continued to exhibit a significant improvement in both VAS and ODI scores at the final follow-up compared with the 3-day postoperative values (P < 0.05). The change in VAS (ΔVAS = -6.08) and ODI (ΔODI = -62.7) of A-PKP group both exceeded the MCID values of -2.2 and -9.5, respectively, indicating clinically meaningful improvements. Similarly, the T-PKP group also exhibited a continuous improvement in ODI scores from the postoperative day 3 follow-up to the final follow-up (P < 0.05). The change in ODI (ΔODI = -59.0) exceeded the MCID threshold of -9.5. Although, VAS scores increased at the final follow-up compared with the values recorded 3 days after surgery (**Table 2**, **Figures 7**, **8**). The change in VAS (ΔVAS = -4.9) exceeded the MCID threshold of -2.2, it still showed a significant reduction in pain compared to preoperative levels.

Furthermore, the rate of adjacent vertebral fractures was significantly lower in the A-PKP group than in the T-PKP group (2 cases, 5.1% vs. 11 cases,18.6%, P < 0.05). The A-PKP group also had a significantly lower incidence of bone cement displacement than the T-PKP group (1 case, 2.5% vs. 9 cases, 20.9%, P < 0.05). Additionally, the incidence of bone cement leakage was significantly lower in the A-PKP group compared to the T-PKP group, though the difference was not statistically significant (8 cases, 20.5% vs. 12 cases, 27.9%, P > 0.05). In the A-PKP group, 8 cases of bone cement leakage occurred, with 4 cases of disc leakage and 4 cases of paravertebral leakage, all of which were asymptomatic. In the T-PKP group, 5 cases of disc leakage and 7 cases of paravertebral leakage occurred, with no obvious symptoms in any case (**Table 2**).

Finally, the logistic regression analysis revealed that only the bone cement distribution score (OR= 0.355, 95% CI 0.171–0.734, P=0.005) was identified as a protective factor following kyphoplasty. In contrast, other factors, including bone cement distribution pattern (OR=0.266, 95% CI 0.037–1.909, P=0.188), bone cement leakage (OR=0.278, 95% CI 0.056–1.373, P=0.116), and bone cement displacement (OR=1.453, 95% CI 0.313–6.749, P=0.633), did not show a statistically significant association with adjacent vertebral fractures (**Table 3**).

**Table 3:** Multivariate logistic regression analysis of the risk factors and AVF.

Variables	B	SE	Wald	OR (95% CI)	p
Bone cement pattern	-1.324	1.006	1.734	0.266 (0.037-1.909)	0.188
Cement distribution score	-1.036	0.371	7.808	0.355 (0.171-0.734)	0.005
Bone cement displacement	-1.280	0.815	2.468	0.278 (0.056-1.373)	0.116
Bone cement leakage	0.374	0.784	0.227	1.453 (0.313-6.749)	0.633

### 3.3 Radiological outcomes

**Table 4** presents the postoperative improvements in fractured vertebra height (AVH, MVH, and PVH) and Cobb angle. No significant differences in AVH, MVH, PVH, WCA, and Cobb angle were observed between the two groups before surgery, 3 days after surgery, and at the final follow-up (P > 0.05). Compared with preoperative measurements, significant differences in AVH, MVH, PVH, WCA, and Cobb angle were observed between the groups 3 days after surgery and at the final follow-up (P < 0.05).

**Table 4:** Comparison of radiographic outcomes of the two groups.

Parameters	A-PKP	T-PKP	t/χ2	p
Anterior vertebral height (mm)				
Preoperative	14.5 ± 5.23	13.9 ± 4.12	0.528	0.599
Postoperative 3 days	19.6 ± 4.14^*^	19.2 ± 3.04^*^	0.567	0.572
Final follow-up	17.9 ± 4.17^*^	17.2 ± 3.15^*#^	0.920	0.361
Middle vertebral height (mm)				
Preoperative	14.1 ± 5.06	13.3 ± 3.83	0.900	0.371
Postoperative 3 days	18.4 ± 3.94^*^	17.6 ± 3.71^*^	0.921	0.360
Final follow-up	16.8 ± 4.30^*^	16.2 ± 3.47^*^	0.797	0.428
Posterior vertebral height (mm)				
Preoperative	22.7 ± 4.18	21.3 ± 3.93	1.623	0.109
Postoperative 3 days	25.4 ± 3.74^*^	24.1 ± 3.99^*^	1.537	0.128
Final follow-up	24.0 ± 3.76^*^	22.5 ± 3.85^*#^	1.890	0.062
Wedge-shape cobb angle (°)				
Preoperative	15.5 ± 4.53	16.8 ± 3.59	-1.484	0.142
Postoperative 3 days	10.8 ± 3.53^*^	11.1 ± 2.61^*^	-0.401	0.690
Final follow-up	12.4 ± 4.14^*^	12.0 ± 2.40^*#^	0.558	0.579
Cobb angle (°)				
Preoperative	20.3 ± 7.54	20.2 ± 8.12	0.800	0.936
Postoperative 3 days	14.9 ± 5.72^*^	14.6 ± 4.74^*^	0.284	0.777
Final follow-up	16.5 ± 6.42^*^	16.2 ± 5.80^*^	0.164	0.870

^*^Compared to pre-operative, p<0.05.

^#^Compared to postoperative 3 days, p<0.05.

In the A-PKP group: the AVH, MVH, PVH, WCA, and Cobb angle significantly from the preoperative values of 14.5 ± 5.23 mm, 14.1 ± 5.06 mm, 22.7 ± 4.18 mm, 15.5 ± 4.53°, and 20.3 ± 7.54°, respectively, to 19.6 ± 4.14 mm, 18.4 ± 3.94 mm, 25.4 ± 3.74 mm, 10.8 ± 3.53°, and 14.9 ± 5.72° at postoperative three day, and to 17.9 ± 4.17 mm, 16.8 ± 4.30 mm, 24.0 ± 3.76mm, 12.4 ± 4.14°, and 16.5 ± 6.42° at final follow-up. No significant differences in AVH, MVH, PVH, WCA, and Cobb angle were observed between postoperative 3 days and the final follow-up (P > 0.05). In the T-PKP group: the AVH, MVH, PVH, WCA, and Cobb angle significantly from the preoperative values of 13.9 ± 4.12 mm, 13.3 ± 3.83 mm, 21.3 ± 3.93 mm, 16.8 ± 3.59°, and 20.2 ± 8.12°, respectively, to 19.2 ± 3.04 mm, 17.6 ± 3.71 mm, 24.1 ± 3.99 mm, 11.1 ± 2.61°, and 14.6 ± 4.74° at postoperative three day, and to 17.2 ± 3.15 mm, 16.2 ± 3.47 mm, 22.5 ± 3.85 mm, 12.0 ± 2.40°, and 16.2 ± 5.80° at final follow-up. No significant differences in MVH and Cobb angle were observed between the postoperative 3 days and the final follow-up (P > 0.05). However, significant differences in AVH, PVH, and WCA were observed between the postoperative 3 days and the final follow-up (P < 0.05).

## 4 Discussion

The present study investigated and compared the clinical and radiographic outcomes of A-PKP versus conventional T-PKP in the treatment of patients with stage I and II KD, particularly focusing on postoperative bone cement displacement. Both techniques showed similar short-term benefits, including satisfactory pain relief, improved vertebral height, and correction of kyphosis. However, A-PKP demonstrated superior long-term outcomes with a reduced incidence of bone cement displacement. The A-PKP group predominantly exhibited a spongy cement distribution pattern, which earned a higher cement distribution score than the T-PKP group. By contrast, the T-PKP group generally exhibited a blocky pattern, which was associated with a lower distribution score and a higher risk of cement displacement.

Advancements in radiological techniques have deepened our understanding of pathogenesis of KD. KD typically progresses gradually, often initiated by minor trauma causing vertebral ischemia and necrosis. Fracture fragments to injure the segmental artery. Trauma may damage the segmental artery, leading to local and subsequent obstruction of blood supply to the vertebra. The ischemia results in avascular necrosis of the vertebra, and as the disease progresses, pseudarthrosis develops within the vertebral body (22). Radiological findings of IVC with marginal sclerosis are characteristic of KD (5, 6), and the thoracolumbar junction is particularly susceptible to this process due to its biomechanical environment (4, 22). Our study further corroborated these findings, revealing that the majority of affected vertebrae in both groups were localized to the T12-L1 region, consistent with prior reports (2, 23, 24). To prevent further collapse and potential compression on the spinal cord or nerves, PVP and PKP are widely accepted as initial treatment options for stage I and II KD, where the posterior vertebral wall remains intact. These procedures offer the benefits of minimal invasiveness, reduced costs, earlier mobilization, and faster rehabilitation through restoration of vertebral height and removal of instability (25). Although both treatments provide similar pain relief outcomes, PKP is associated with a lower incidence of bone cement leakage, superior restoration of vertebral height, and better correction of kyphosis (10, 26). Through a meta-analysis, Xiang et al. (27) revealed that unilateral balloon kyphoplasty is as effective as bilateral balloon kyphoplasty for the treatment of OVCFs, as evidenced by their clinical and radiological results. Labarbela et al. (28) conducted a finite element analysis and found that PKP is more effective than PVP in reducing adjacent endplate stress and fracture risk, thereby making it the preferred treatment choice for thoracolumbar OVCFs. Additionally, gas or fluid present in the IVC in KD suggests that bone cement can flow unimpeded and distribute symmetrically. Consequently, PKP through a unilateral approach was selected for this study.

Conventional PKP primarily relies on the injection of PMMA into the IVC, which alleviates pain probably through mechanical stabilization as well as potential chemical toxicity or thermal necrosis of the adjacent tissues and nerve endings (29). However, because of the presence of a thick fibrocartilaginous membrane surrounding the IVC, the injected cement typically forms a blocky pattern, limiting its distribution and interdigitation with the surrounding cancellous bone (5, 13, 30). According to several studies, a diffused cement pattern yields better clinical and radiographic outcomes than a blocky pattern (13, 20, 31). Lee et al. (32) observed that a less distributed, blocky cement pattern fails to interlock effectively with the surrounding cancellous bone, functioning more as a space-occupying material rather than providing mechanical support. Additionally, the uncemented cancellous bone situated between the endplate and the injected cement becomes vulnerable to fractures, potentially due to the stress-shielding effect of the cement. This weakened interlocking structure increases the risk of bone cement displacement and fractures (33), which can lead to recurrent pain, further kyphotic deformity, and catastrophic complications (14). The incidence of bone cement displacement in PKP is estimated to range between 5% and 6% (14, 34), making it a critical complication to address. Several methods have recently been employed to improve bone cement distribution and reduce the risk of bone cement displacement. Wang et al. (12) demonstrated that PKP via the transverse process-pedicle approach provided more symmetrical cement distribution than the traditional approach. Zhong et al. (35) introduced a rotary cutter to disrupt the dense fibrocartilaginous membrane surrounding the IVC, resulting in favorable outcomes. Zhong et al. (36) further innovated with a hollow pedicle screw combined with kyphoplasty, which improved the stability of bone cement and reduced the incidence of bone cement displacement. Wang et al. (37) designed a novel bone cement screw system combined with PVP to treat anterior cortex defects in KD patients, achieving promising therapeutic results. Distefano et al. (38) proposed the Stent-screw-assisted internal fixation technique (SAIF), which allows for vertebral body reconstruction and restoration of axial load, particularly in cases with large osteonecrotic clefts.

Compared to conventional T-PKP, A-PKP employed a novel approach using a curved-tipped Kirschner needle to create multiple puncture points on the fibrocartilaginous membrane surrounding the IVC. This technique significantly the interdigitation of bone cement with the surrounding cancellous bone. Additionally, approximately 1ml of late-phase wire-drawing cement was injected near the anterior cortex to block potential pathways for bone cement displacement. This method significantly reduced the incidence of postoperative cement displacement during follow-up (2.5%; P < 0.05). The injected cement permeated the IVC and penetrated through the puncture points in the fibrocartilaginous membrane, establishing contact with both upper and lower endplates. These factors notably improved the anchoring stability of the bone cement within the vertebral body. Moreover, the initial injection of bone cement into the anterior cortex effectively blocked displacement pathways. This “anterior sealing, posterior anchoring” strategy may account for the reduced risk of postoperative cement displacement. This improved cement interlocking and minimized the risk of fractures in uncemented cancellous bone, ultimately producing a spongy cement pattern with a high cement distribution score (P < 0.05). Liu et al. (20) confirmed that diffuse bone cement pattern with a score of 10 appears to be the best balance between preventing re-collapse and new fractures. The 12-score method is less effective in predicting new fractures than re-collapse. Our findings are consistent with theirs, as the A-PKP group achieved a balanced cement distribution score (10.0 ± 1.17; P < 0.05) with a lower incidence of adjacent vertebra fractures (P < 0.05). At the same time, our logistic regression analysis revealed that the bone cement distribution score was identified as a protective factor of adjacent vertebra fractures following kyphoplasty. It is possible that the uniform distribution of bone cement effectively transmits stress between the vertebrae and intervertebral discs, thereby reducing the stress on adjacent vertebrae and subsequently decreasing the incidence of adjacent vertebra fractures. Next, we plan to conduct a three-dimensional finite element analysis to investigate the relationship between bone cement distribution patterns and the stress on adjacent vertebrae after A-PKP, further validating our hypothesis. Ye et al. (39) demonstrated that insufficient cement filling is associated with chronic lower back pain. The A-PKP group exhibited better improvement in VAS and ODI scores compared with the T-PKP group during follow-up assessments (P < 0.05). More diffuse distribution pattern of bone cement in the A-PKP group might create larger contact area with the vertebral bone compared to the confined distribution in T-PKP. The increased contact area enhances the chemical toxicity and thermal effects of the cement, effectively destroying pain-related nerve endings within the vertebral body. Additionally, this distribution pattern significantly strengthens vertebral biomechanical stability and eliminates intravertebral pseudarthrosis. Both groups exhibited significant improvements in vertebral height and kyphosis angle following surgery, indicating that A-PKP can restore vertebral height and improve kyphosis, similar to conventional T-PKP However, minor deterioration in these parameters was observed at the final follow-up due to the natural progression of osteoporosis (40) (P > 0.05), which is consistent with Chen’s findings (13).

The duration of the A-PKP procedure was marginally longer than that of the T-PKP procedure, primarily due to the additional step of creating multiple puncture points using the Kirschner needle (P < 0.05). Sun et al. (19) 4–6 mL of bone cement is sufficient for rapid pain relief in cases of mild-to-moderate OVCFs at a single thoracolumbar level. Although an increased cement volume generally increases the risk of cement leakage, our study demonstrated that despite a higher volume of cement in the A-PKP group, the incidence of cement leakage was lower (P > 0.05). This may be attributed to the use of the curved-tipped Kirschner needle during A-PKP, which creates multiple puncture points on the fibrocartilaginous membrane surrounding the IVC. This technique facilitates the diffusion of bone cement into the surrounding cancellous bone, reducing the injection pressure and consequently lowering the incidence of cement leakage. The Bone cement distribution score (OR = 0.355, 95% CI 0.171–0.734, P = 0.005) was a significant protective factor, likely due to its role in enhancing vertebral stability and reducing stress concentration, thereby lowering the risk of AVF. In contrast, the bone cement distribution pattern (OR = 0.266, 95% CI 0.037–1.909, P = 0.188) showed no significant effect, potentially due to its limited ability to reflect stress transfer. Bone cement displacement (OR = 1.453, 95% CI 0.313–6.749, P = 0.633) and bone cement leakage (OR = 0.278, 95% CI 0.056–1.373, P = 0.116) did not significantly impact AVF risk, possibly due to the small degree of displacement and the minimal effect of leakage, which occurred primarily in the paravertebral area and did not substantially alter vertebral load distribution.

For the successful application of the A-PKP technique, several critical considerations should be followed. (1) Patient selection: Care should be taken to exclude patients with posterior vertebral wall defects to avoid the risk of cement leakage into the spinal canal. (2) Fibrocartilaginous Membrane Disrupting: Employing a curved-tipped Kirschner needle to disrupt the dense fibrocartilaginous membrane around the IVC enhances the diffusion and anchoring of the cement within the cancellous bone. (3) Bone cement injection: A three-stage infusion process must be employed to optimize cement placement.

This study has several limitations that should be considered when interpreting the findings. First, the retrospective design may introduce inherent biases, including selection bias. Second, the small sample size and single-center design limit the generalizability of the results. Additionally, the relatively short follow-up period raises concerns, particularly for a disease that is prone to late complications. Despite these limitations, the study provides valuable insights into the clinical outcomes of a novel technology for stage I and II KD. Future research will focus on a prospective randomized controlled trial to validate these findings and assess the long-term outcomes of this technology in a broader, more diverse population.

## 5 Conclusion

A-PKP is a safer and more effective alternative for treating stage I and II of KD patients. It effectively alleviates patients’ symptoms, promotes better cement diffusion, and prevents bone cement displacement compared with the conventional T-PKP approach. However, to further validate these findings and assess the long-term outcomes, larger, prospective studies are needed.

## Data Availability

The raw data supporting the conclusions of this article will be made available by the authors, without undue reservation.
